# Primary Testicular Tuberculosis Presenting as a Scrotal Emergency: A Report of a Rare Case

**DOI:** 10.7759/cureus.87951

**Published:** 2025-07-14

**Authors:** Haritha Shahnaz Masthan, Sundeep Selvamuthukumaran, Pola Govardhan Kumar, B V Sreedevi, Raakesh Madhavan R S

**Affiliations:** 1 General Surgery, Sree Balaji Medical College and Hospital, Chennai, IND; 2 General Surgery, Sree Balaji Medical College and Hospital, Chennai, India

**Keywords:** extrapulmonary tb, genitourinary tuberculosis, mycobacterium tuberculosis, pyocele, scrotal abscess, scrotal swelling, testicular tuberculosis

## Abstract

Tuberculosis (TB) continues to pose a major health challenge worldwide, particularly in regions where the disease is endemic, such as India. Although the lungs are the most commonly affected, TB can also involve other organs, including the genitourinary system. Testicular involvement is extremely uncommon and can clinically resemble more frequent scrotal conditions like pyocele or epididymo-orchitis. We present the case of a 50-year-old male who developed unilateral scrotal pain and swelling, initially suggestive of an acute infection. Emergency scrotal exploration uncovered an abscess with necrotic testicular tissue. Histopathological examination confirmed testicular tuberculosis. The patient responded well to anti-tuberculous treatment, emphasizing the need to include TB in the differential diagnosis of atypical scrotal swellings, especially in endemic areas.

## Introduction

Tuberculosis (TB) continues to be a major global health issue, with India contributing significantly to the global burden of disease. According to Al-Hashimi and Said, extrapulmonary TB represents a notable proportion of cases, with genitourinary involvement being one of the less common manifestations [1]. Although pulmonary TB is the most prevalent form, disseminated TB can involve various organs, including the kidneys, prostate, seminal vesicles, and, rarely, the testicles [2].

Ramana emphasized the rising threat of disseminated TB in the era of multidrug-resistant tuberculosis, further increasing the likelihood of atypical presentations [3]. The latent TB infection (LTBI) rate has been re-estimated to be extremely high in countries like India, where around 26% of the global LTBI burden resides [4]. TB primarily spreads via respiratory droplets [5], but in some cases, hematogenous or lymphatic spread can lead to extrapulmonary involvement, including the genitourinary tract [6].

Testicular TB is exceedingly rare and often presents as unilateral testicular swelling or a scrotal mass, making it difficult to differentiate from other more common scrotal pathologies such as pyocele, epididymo-orchitis, or even testicular tumors [7]. Pediatric cases have also been reported, further broadening the clinical spectrum of this entity [8]. Hadadi et al. documented the challenges of diagnosing isolated testicular TB due to its overlapping features with more frequent urological conditions [9].

## Case presentation

**Figure 1:**
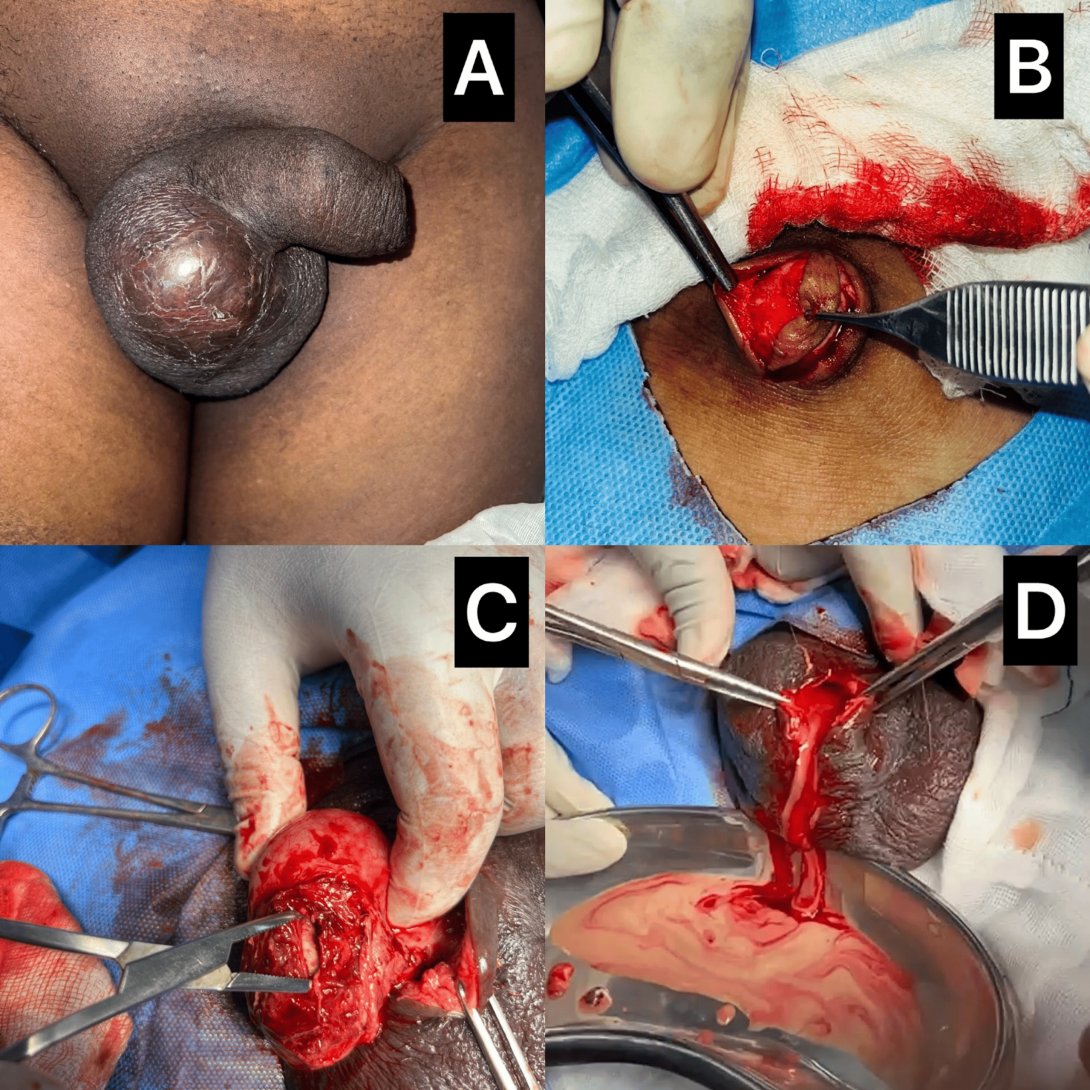
Sequential intraoperative and preoperative findings of testicular tuberculosis (A) Preoperative clinical image showing tense, smooth, right-sided scrotal swelling with shiny overlying skin and no obvious sinus or discharge. (B) Intraoperative exposure reveals a collar-stud abscess tracking into the subcutaneous plane during initial incision. (C) Operative dissection showing inflamed and necrotic testicular tissue with surrounding pus pockets suggestive of tuberculous orchitis. (D) Thick yellowish-white caseous material draining from the abscess cavity, typical of tubercular pathology.

A 50-year-old Indian male presented to the general surgery outpatient department with complaints of progressive right-sided scrotal swelling and intermittent pain over the past four weeks. The pain was dull in nature, localized, and associated with a low-grade evening-predominant fever. There was no history of urinary symptoms, such as dysuria, frequency, or hematuria, nor was there any recent trauma, sexual activity, or systemic illness. His medical and family history were non-contributory, and he had no known exposure to tuberculosis or travel to TB-endemic zones outside of his local environment. On general physical examination, the patient was conscious, alert, and afebrile at the time of evaluation. His vital signs were within normal limits: pulse rate was 80 beats per minute, blood pressure was 130/70 mmHg, respiratory rate was 20 breaths per minute, and SpO₂ was 98% on room air. Local examination of the scrotum revealed a 6 × 5 cm right-sided scrotal swelling that was fluctuant, erythematous, tender, and non-transilluminant, with associated excoriation of the overlying skin (Figure 1A). The right testis was not separately palpable, while the left testis and cord structures were unremarkable. Additionally, palpable, discrete right inguinal lymph nodes were noted.

Laboratory tests revealed mild leukocytosis with elevated ESR and CRP. Other investigations, including hemoglobin, renal and liver function tests, and serology, were unremarkable (Table 1). Scrotal ultrasonography showed a hypoechoic lesion in the upper pole of the right testis with peripheral low vascularity, raising suspicion for an abscess or infective process such as pyocele.

**Table 1:** Baseline laboratory investigations with reference ranges and interpretation

Test	Patient Value	Reference Range	Interpretation
Hemoglobin	13.2 g/dL	13.5 – 17.5 g/dL (male)	Mildly low-normal
Total Leukocyte Count (TLC)	11.5 × 10⁹/L	4.0 – 11.0 × 10⁹/L	Mild leukocytosis (suggests infection)
Platelet Count	350 × 10⁹/L	150 – 400 × 10⁹/L	Normal
Erythrocyte Sedimentation Rate	52 mm/hr	< 20 mm/hr	Elevated (indicates inflammation)
C-Reactive Protein (CRP)	Positive (24 mg/L)	< 6 mg/L	Elevated (acute phase reactant)
Random Blood Sugar	118 mg/dL	70 – 140 mg/dL	Normal
Serum Creatinine	0.9 mg/dL	0.6 – 1.2 mg/dL	Normal renal function
Liver Function Tests (LFTs)	Within normal limits	—	Normal
HIV, HBsAg, Anti-HCV	Non-reactive	Negative	Rule out immunocompromise/coinfection
Chest X-ray	No abnormalities	Clear lung fields	No pulmonary TB

Based on clinical and radiological findings, a diagnosis of right-sided scrotal pyocele was considered, and emergency surgical exploration was undertaken after obtaining informed consent and fitness for anesthesia. Intraoperative findings included approximately 20 ml of purulent material in the subcutaneous plane, tracking to the necrotic, dusky right testis. The appearance mimicked a collar-stud abscess (Figures 1B-1D). The involved tissue was excised and sent for histopathological evaluation.

**Figure 2:**
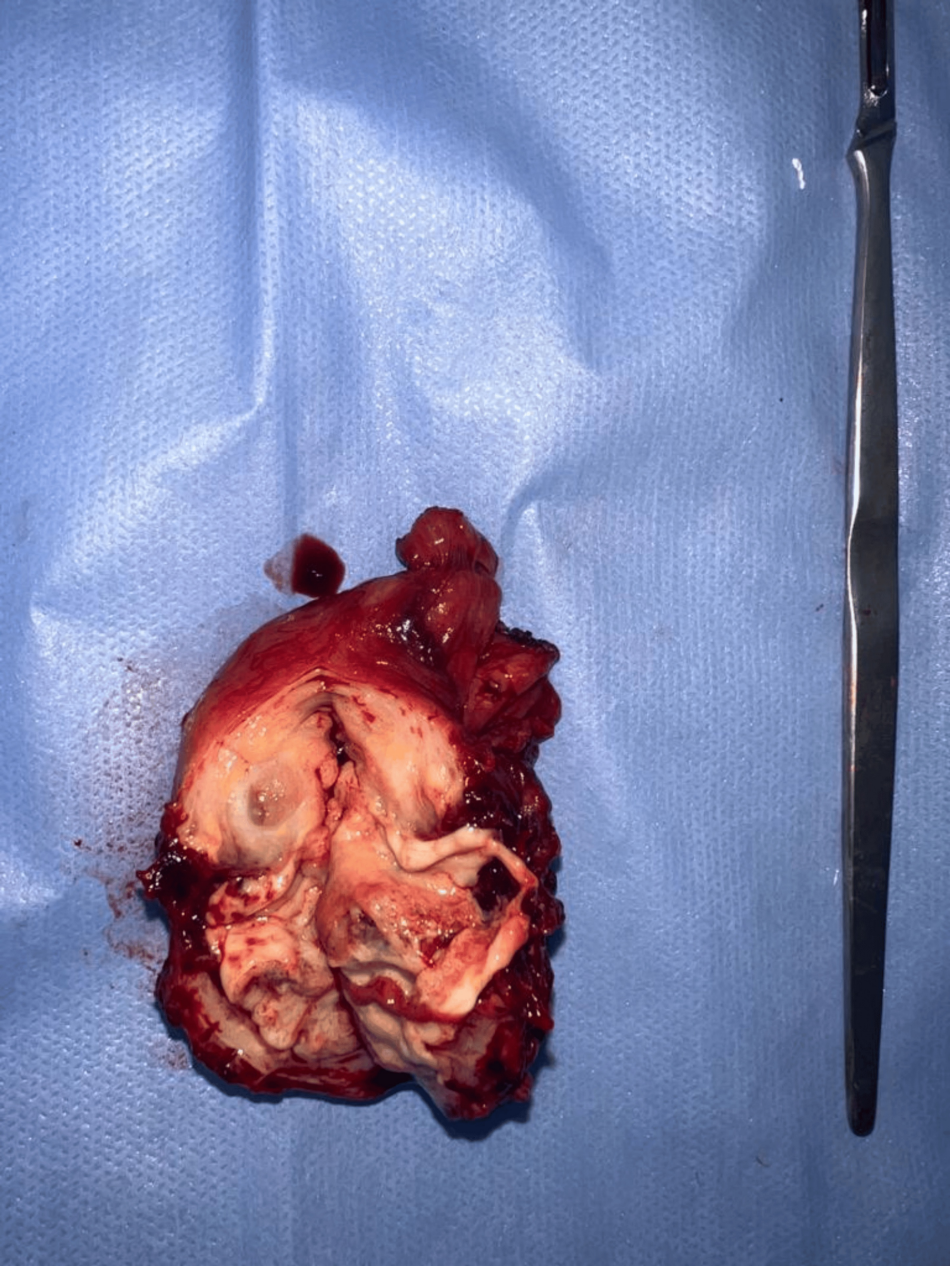
Cut section of resected right testis

Histopathological examination revealed granulomatous inflammation with caseous necrosis, epithelioid cells, and Langhans giant cells, findings pathognomonic of tuberculosis. The diagnosis of testicular tuberculosis was thus confirmed. Postoperatively, the patient was initiated on first-line anti-tubercular therapy comprising rifampicin (600 mg), isoniazid (300 mg), pyrazinamide (2 g), and ethambutol (1.3 g) daily for two months, followed by continuation therapy with rifampicin and isoniazid for the next four months. The patient showed significant clinical improvement, with resolution of inflammation and proper healing of the scrotal wound. A follow-up ultrasound at three months showed no signs of recurrence or residual disease. The intraoperative image shows the bisected right testis post-orchidectomy (Figure 2). The cut surface reveals areas of necrosis and multiple loculated pus pockets, consistent with chronic granulomatous inflammation and abscess formation characteristic of tuberculous orchitis.

**Figure 3:**
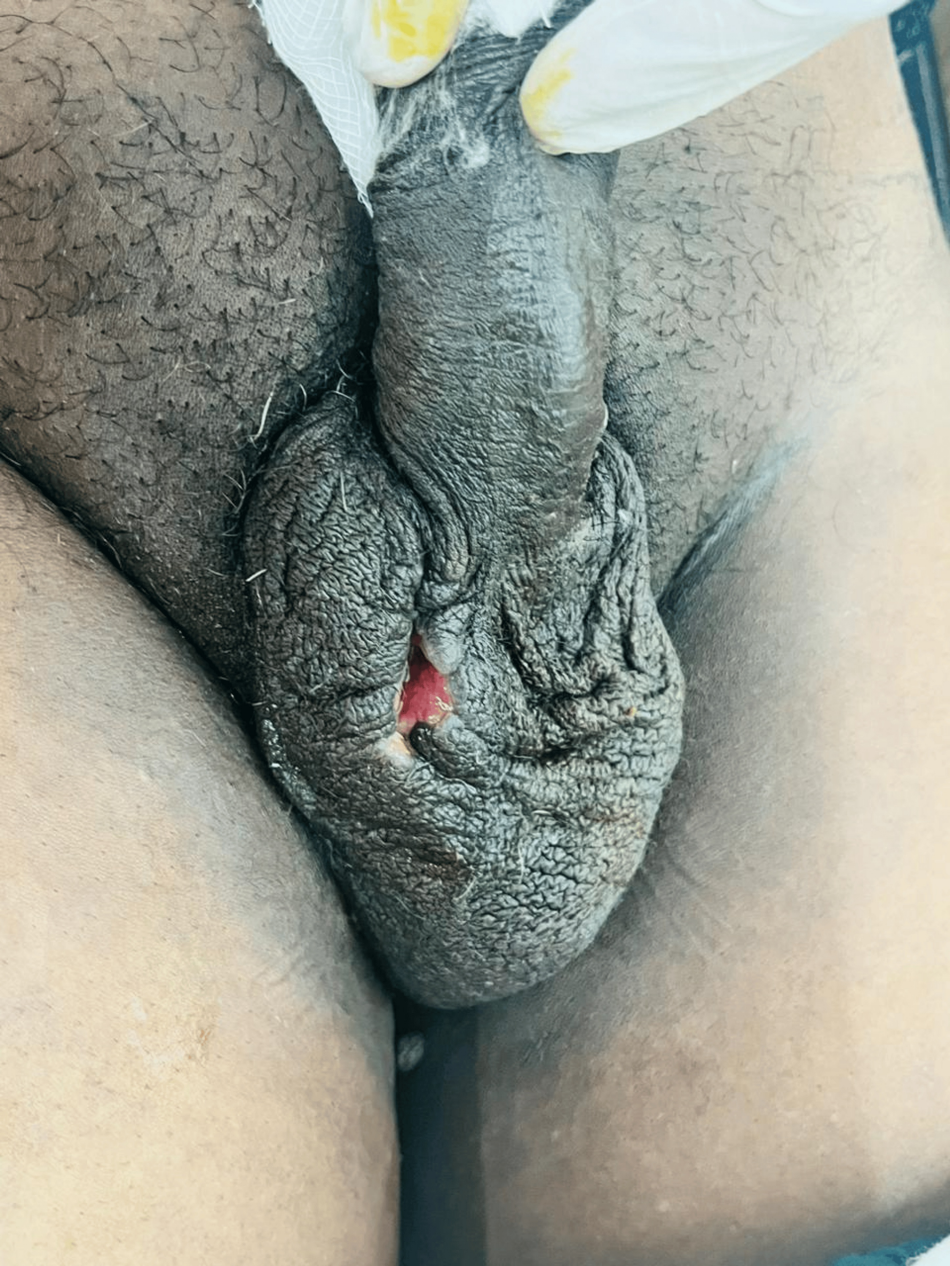
Four-month postoperative follow-up after anti-tubercular therapy

At four-month follow-up after completion of anti-tubercular therapy, the patient was clinically stable with no systemic symptoms or signs of recurrence. Local examination revealed a healing scrotal ulcer at the previous surgical site with healthy granulation tissue and no signs of active infection or discharge (Figure 3). The patient reported good adherence to therapy and resolution of initial symptoms, indicating an effective response to the six-month ATT regimen.

## Discussion

Testicular tuberculosis (TB) represents a rare and often overlooked form of genitourinary tuberculosis, accounting for less than 3% of all extrapulmonary TB cases [1]. Al-Hashimi and Said emphasized that this form can easily be misdiagnosed due to its clinical overlap with other common scrotal pathologies [1]. Although genitourinary TB was first systematically described decades ago, its clinical relevance persists, especially in TB-endemic countries where atypical presentations are common. The testis may become involved through hematogenous dissemination or retrograde extension from adjacent organs such as the prostate and seminal vesicles [2]. Muttarak and Peh demonstrated through imaging case reports that testicular TB often mimics pyogenic epididymo-orchitis or neoplasms, which complicates the diagnosis [2].

Disseminated tuberculosis and extrapulmonary manifestations, such as genitourinary TB, are increasingly reported in the context of immunosuppression and multidrug-resistant TB (MDR-TB). Ramana highlighted the risk of MDR-TB leading to unusual manifestations like bilateral renal abscess and hydronephrosis, reminding clinicians to consider TB in persistent or unresolving urological infections [3]. Houben and Dodd further estimated that India harbors a significant proportion of the global latent TB burden, increasing the likelihood of such extrapulmonary forms in the general population [4]. TB typically spreads via the inhalation of aerosolized droplets [5], but extrapulmonary spread, especially through lymphatic or hematogenous routes, may involve virtually any organ, including the testes [6]. Viveiros et al. presented a case of isolated testicular TB without pulmonary involvement, consistent with the presentation in our patient, underscoring the unpredictable dissemination pattern of Mycobacterium tuberculosis [6].

Testicular TB may clinically mimic epididymo-orchitis, pyocele, scrotal abscess, or even neoplasm, making accurate diagnosis challenging. Pediatric cases, such as the one reported by Gurubacharya and Gurubacharya, confirm that TB can affect all age groups and often presents without classic systemic signs [7]. Hadadi et al. also reported a case where isolated testicular TB was misdiagnosed as a tumor until confirmed by histopathology, a common scenario when the presentation is unilateral and painless [8]. Similarly, Hassan and colleagues described bilateral testicular tuberculomas in a patient with otherwise unremarkable history, indicating that the condition can present in both unilateral and bilateral forms [9]. Differentiating testicular tuberculosis from other causes of scrotal swelling is critical due to overlapping presentations and imaging features. A comparative overview of clinical, radiological, and pathological distinctions is summarized in Table 2.

**Table 2:** Comparison of testicular tuberculosis with the common differential diagnoses of scrotal swelling

Condition	Clinical Features	Ultrasound Findings	Histopathology	Management
Testicular Tuberculosis	Chronic pain/swelling, systemic symptoms, often no urinary signs	Hypoechoic lesion with peripheral vascularity, abscess	Granulomatous inflammation with caseation	Anti-tubercular therapy ± surgery
Epididymo-orchitis	Painful swelling, fever, dysuria	Enlarged epididymis, increased blood flow	Acute inflammation, neutrophilic infiltrate	Antibiotics
Testicular Tumor	Painless firm mass, no systemic symptoms	Solid intratesticular mass, usually hypervascular	Malignant cells, no granulomas	Orchiectomy ± chemo/radiotherapy
Pyocele	Painful, fluctuant scrotal swelling	Fluid with echogenic debris, septations	Pus collection, no granulomas	Incision and drainage ± antibiotics

Das et al. explored the incidence and pathological aspects of genitourinary TB in India, noting that despite being the third most common site of extrapulmonary TB, diagnosis is frequently missed due to the insidious nature of the disease [10]. Matsumura et al. reported renal TB mimicking hydronephrosis, reflecting how TB can take on the guise of more benign urological conditions and delay diagnosis [11]. Gulati et al., in their report on pediatric nephrotic syndrome, emphasized the importance of screening for TB in atypical or steroid-resistant nephrotic syndromes in endemic areas [12].

Testicular TB can also mimic malignancy, as in the case reported by Cho et al., where a young athlete underwent unnecessary evaluation for suspected testicular cancer, which later proved to be TB of both the testis and prostate [13]. Kim et al. reviewed the sonographic findings of tuberculous epididymo-orchitis, showing that such lesions often appear as hypoechoic masses with low vascularity, mimicking tumors or abscesses but lacking definitive diagnostic features without biopsy [14]. Abraham et al. reinforced this diagnostic challenge by documenting a healthy young man with a testicular mass that appeared malignant but was histologically confirmed to be tuberculosis only after surgical exploration [15].In our case, imaging revealed a hypoechoic lesion suggestive of a pyocele or abscess, and intraoperative findings revealed necrotic testicular tissue with pus. The final diagnosis of testicular TB was established only after histopathological examination demonstrated epithelioid granulomas with Langhans giant cells and central caseation, which are hallmarks of TB [1,2,14]. This case supports the importance of histology in atypical scrotal swellings, particularly when clinical and radiologic findings are inconclusive.

Anti-tubercular therapy remains the cornerstone of treatment, and most cases resolve with a standard six-month regimen. Surgical exploration is warranted in cases with abscess formation, necrosis, diagnostic uncertainty, or failure of medical therapy [1,13]. Early recognition and targeted treatment can prevent complications such as infertility and unnecessary orchiectomy. Thus, clinicians in endemic regions should always include TB in the differential diagnosis of any persistent, atypical, or treatment-resistant scrotal pathology [3,5,6].

## Conclusions

Testicular tuberculosis, though rare, should not be overlooked in patients presenting with persistent or atypical scrotal swelling, especially in TB-endemic countries. Misdiagnosis can lead to inappropriate treatment or unnecessary surgical interventions. This case underscores the importance of maintaining a high index of suspicion, particularly in cases unresponsive to antibiotics, and emphasizes the role of histopathology in diagnosis. Early initiation of anti-tubercular therapy can lead to favorable outcomes and prevent complications such as infertility. A multidisciplinary approach involving clinical, imaging, and pathological correlation is essential for the accurate diagnosis and effective management of this rare presentation.
